# Gastrodia elata Blume alleviates L-DOPA-induced dyskinesia by normalizing FosB and ERK activation in a 6-OHDA-lesioned Parkinson’s disease mouse model

**DOI:** 10.1186/1472-6882-14-107

**Published:** 2014-03-20

**Authors:** Ah-Reum Doo, Seung-Nam Kim, Dae-Hyun Hahm, Hye Hyun Yoo, Ji-Yeun Park, Hyejung Lee, Songhee Jeon, Jongpil Kim, Seong-Uk Park, Hi-Joon Park

**Affiliations:** 1 Studies of Translational Acupuncture Research (STAR), Acupuncture & Meridian Science Research Center (AMSRC), Kyung Hee University, 1 Hoegi-dong, Dongdaemoon-gu, Seoul 130-701, Republic of Korea; 2 Department of Pharmacy, College of Pharmacy, Hanyang University, Ansan-si, Gyeonggi-do 426-791, Republic of Korea; 3 Dongguk University Research Institute of Biotechnology, Dongguk University, 3-26, Pil Dong, Choong-Gu, Seoul 100-715, Republic of Korea; 4 Department of Neurology and Cardiology of Korean Medicine, College of Korean Medicine, Kyung Hee University, Seoul 134-727, Republic of Korea; 5 Stroke and Neurological Disorders Center, Kyung Hee University Hospital at Gangdong, 149 Sangil-dong, Gangdong-gu, Seoul 134-727, Republic of Korea

**Keywords:** Gastrodia elata blum, Parkinson’s disease, Levodopa-induced dyskinesia, ERK1/2, FosB

## Abstract

**Background:**

Gastrodia elata Blume (GEB), commonly used medicinal herb, has been reported as a promising candidate for neurodegenerative diseases such as Parkinson’s disease. The dopamine precursor, L-3,4-dihydroxyphenylalanine (L-DOPA), is the gold-standard drug for Parkinson’s disease, but long-term treatment results in the L-dopa-induced dyskinesia (LID). This study was undertaken to examine the beneficial effects of GEB on L-DOPA induced dyskinesia in 6-hydroxydopamine (6-OHDA)-induced experimental Parkinsonism.

**Methods:**

We tested the effects of GEB on LID in 6-hydroxydopamine hydrochloride-hemiparkinsonian mice. To analyze the dyskinetic anomalies, we measured abnormal involuntary movement (AIM). Immunohistological analyses of pERK and FosB expressions in the striatum are performed to explore the mechanism of GEB on LID.

**Results:**

The finding of this study demonstrated that GEB (200, 400 and 800 mg/kg) alleviated L-dopa induced AIMs in a dose-dependent manner. In each integrative AIM subtype analysis, we also found that the GEB (400 and 800 mg/kg) treatment decreased L-DOPA-induced axial, limb, orolingual, and locomotive AIMs compared to the LID group. In addition, GEB normalized the abnormal LID-induced increase of pERK1/2 and FosB, the immediate early genes of LID in the striatum.

**Conclusions:**

In conclusion, our results provide a novel insight into the pharmacological actions of GEB that could have a benefit for PD patients through the reduction of LID.

## Background

Parkinson’s disease (PD), which afflicts 1-2% of the population over 65 years of age, is the second most common neurodegenerative disorder. PD arises from the striatal depletion of dopamine (DA) [1-3]. This characteristic has led to the use of dopamine agonists, including 3,4-dihydroxy-L-phenylalanine (L-DOPA), to replace dopamine and enhance synaptic DA transmission [4,5]. Although dopaminergic drugs are highly effective agents for the symptomatic treatment of PD [4-8], patients who have undergone long-term L-DOPA administration experience adverse effects, including motor fluctuations and dyskinesia [9-11]. Increasing reports regarding these adverse effects of L-DOPA have led to a backlash among some PD patients who fear these side effects more than they value the therapeutic benefit of L-DOPA treatment [12]. Dyskinesis is a common complication of L-DOPA treatment with an incidence of approximately 10% of PD patients per year. Some 40-50% of PD patients who undergo L-DOPA treatment for 4–6 years developed dyskinesia, and the incidence increased to 90% in patients treated for 10 years [6,13,14].

Previous studies reported that L-DOPA-induced dyskinesia (LID) in PD is mediated by alterations in basal ganglia activity [15,16]. It is believed that chronic treatment with L-DOPA (with its short half-life, requirement for pulsatile delivery to the brain and subsequent stimulation of postsynaptic striatal neurons with dopamine) is a key factor leading to the induction of dyskinesia in PD patients [2,17].

Gastrodia elata Blume (GEB) belongs to the Orchidaceae family and is one of the most popular herbs used by patients across different clinical settings in Asia. It has been used in the treatment of many conditions, such as convulsion, ischemia, dementia, tremors, and vertigo [18-20]. A number of studies have been conducted to investigate possible effects of GEB on brain function. For example, GEB has been found to inhibit glutamate-induced apoptosis in neuroblastoma cells [21], kainite-induced neuronal damage [22], and MPP ^+^ −induced cytotoxicity in SH-SY5Y cells [23]. It was also found to protect against neuronal cell damage following transient global brain ischemia [24]. GEB contains various biologically active components [25] that may improve neuronal cell viability through inhibition of apoptosis [24,26]. This may also be the mechanism by which GEB treatment protects neuronal cells damaged by transient brain ischemia [26]. While many studies have shown the potential utility of GEB in PD treatment, no experiments have examined the effects of GEB on the amelioration of adverse L-DOPA effects in PD models.

In this study, we used a paradigm which is representative of clinical L-DOPA use: intermittent delivery of a constant dose of L-DOPA to mice unilaterally lesioned with 6-OHDA. To assess the value of GBE in PD, we initially investigated the anti-dyskinetic effects of the GEB extract in an LID mouse model.

## Methods

### Animals

Nine-week-old male C57Bl/6 mice (Central Lab. Animal Inc., Seoul, Republic of Korea), weighing 23–26 g each, were used in all experiments. The animals were housed under a 12 h light/12 h dark cycle with ad libitum access to food and water.

All experiments were approved by the Kyung Hee University Animal Care Committee for animal welfare [KHUASP(SE)-09-046] and maintained in strict accordance with Guidelines of the NIH and Korean Academy of Medical Sciences for animal care and use of laboratory animals at Kyung Hee University, Republic of Korea.

### Unilateral 6-OHDA lesion

Mice were anesthetized with a mixture of tiletamine and zolazepam (30 mg/kg; Zoletil 50, Virbac, France) and xylazine (10 mg/kg; Rompun, Bayer Korea, Republic of Korea) in physiological saline and mounted in a stereotaxic frame with a mouse adaptor (Stoelting Co., Wood Dale, IL, USA). A solution of 6-OHDA-HCl (3.0 μg/μL) was prepared in saline containing 0.02% ascorbic acid (Sigma-Aldrich, St. Louis, MO, USA). Each mouse received two unilateral injections of 6-OHDA-HCl (2 μL) into the right striatum, as previously described, according to the following coordinates: (1) AP +1.0 mm, ML −2.1 mm, DV −3.2 mm; and (2) AP +0.3 mm, ML −2.3 mm, DV −3.2 mm (total 2 sites × 2 μL). For control mice, two intrastriatal injections of saline were given at the same coordinates. Each injection was performed at a rate of 0.5 μL/min using a glass capillary with an outer diameter of 50 μm attached to a Hamilton syringe. After the injection, the capillary was left in place for an additional 3 min before being retracted slowly [13]. Mice were allowed to recover from surgery for 15 days in their home cages prior to the start of any experimental procedure.

### Drugs and experimental design

**Figure 1:**
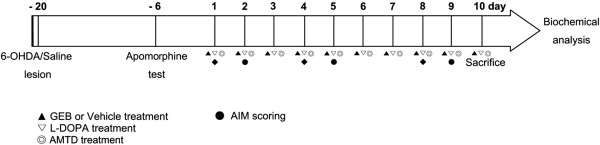
**Schematic representation of experimental design.** Numerals within the time course arrow denote the number of days after the start of chronic treatment with L-DOPA, GEB (200, 400, and 800 mg/kg), AMTD (40 mg/kg), or vehicle. Contralateral turning behavior was tested 14 days later, after apomorphine injection. Three weeks after the introduction of 6-OHDA lesions, mice were administered with L-DOPA, GEB, or AMTD daily for 10 days. AIMs scores were assessed on days 2, 5, and 9 after initial treatment.

GEB (Product #: 5413, Lot#: 142311) manufactured by Sunten Pharmaceutical (Taipei, Taiwan) have been approved as herbal medicines by Taiwan Food and Drug Adminstration (TFDA). TFDA accepted GEB manufacturing process, efficacy and safety assurance, quality control method. Extract of GEB was deposited by Kyung Hee University herbarium (Deposit #: KHH-G-0028). After a period of recovery from the 6-OHDA lesions, mice were divided randomly into experimental groups and administered L-DOPA (20 mg/kg, intraperitoneally) and daily drug treatments for 10 days. Daily drug treatments consisted of oral administration of 200, 400, and 800 mg/kg of the GEB extract or 40 mg/kg of amantadine (AMTD; Sigma-Aldrich, St. Louis, MO, USA). L-DOPA was freshly dissolved in physiological saline and combined with 12 mg/kg of benserazide (Sigma-Aldrich, St. Louis, MO, USA), as previously described [27]. The GEB extract and AMTD were dissolved in distilled water by sonication in an ultrasonic water bath for 3 h. The GEB extract was administered at 120 min prior to the L-DOPA injection and AMTD was administered at 100 min prior. These experimental protocols have been reported previously to lead to the development of LID and maximal anti-dyskinetic potency of AMTD in the same mouse model used in this study [13]. The experimental procedure is also shown in Figure 1. The experimental groups were as follows (n = 7-10 in each group): (1) 6-OHDA group, 6-OHDA + vehicle + vehicle; (2) L-DOPA group, 6-OHDA + L-DOPA + vehicle; (3) GEB200 group, 6-OHDA + L-DOPA + GEB extract 200 mg/kg; (4) GEB400 group, 6-OHDA + L-DOPA + GEB extract 400 mg/kg; (5) GEB800 group, 6-OHDA + L-DOPA + GEB extract 800 mg/kg; and (6) AMTD group, 6-OHDA + L-DOPA + AMTD 40 mg/kg.

### Abnormal involuntary movement (AIM) score

We used an AIM assessment method to examine LID in the mice, as previously described [27-30]. To quantify LID, each mouse was placed in a separate cage for a blinded observer to assess abnormal behaviours. The observations were made individually every 20 min from 20 to 140 min after the injection of L-dopa or vehicle. Over the course of the experiment, AIM scores of the mice were evaluated at 2, 5, and 9 days after the first L-DOPA injection. AIM scores were classified based on topographic distribution into four different subtypes: (1) axial AIM, defined as contralateral twisted posturing of the upper body and neck; (2) limb AIM, indicated by repetitive, rhythmic jerky movements or dystonic posturing of the forelimb on the side contralateral to the lesion; (3) orolingual AIM, characterized by orofacial muscle twitching, empty masticatory movements and contralateral tongue protrusion; and (4) locomotive AIM, defined as increased locomotion with contralateral side bias. Each of these four subtypes was scored on a severity scale from 0 to 4 (0, absent; 1, present for less than half of the observation time; 2, present for more than half of the observation time; 3, continually present but suppressible by outer stimuli; 4, continually present and not suppressible). A total AIMS score was calculated for each mouse by combining each of the four individual dyskinesia scores. The assessments were performed in a blind manner.

### Western blotting

Within 30 min after the last L-DOPA injection, the striatum was quickly dissected and lysed in lysis buffer (2.5 M NaCl, 1 M TrisHCl (pH 7.5), 0.5 M sodium diphosphate, 1 M NaF, 0.5 M EDTA, 0.5 M Na3VO4, and 10% Triton X-100). Lysates were cleared by centrifugation and the protein concentration was determined. Some 20 μg of total protein was separated by 10% (w/v) SDS-PAGE and transferred to a PVDF membrane. The membrane was shaken in Tris-buffered saline containing 0.1% Tween-20 for washing. The membrane was shaken for 1 h at room temperature in 5% (w/v) skim milk to block nonspecific signals and incubated overnight with primary antibodies at 4°C. The following primary antibodies were used in this study: β-actin (Santa Cruz Biotechnology Inc., Dallas, TX, USA), Phospho-ERK1/2, ERK1/2, and FosB (Cell Signaling Technology, Danvers, MA, USA). The membrane was shaken for 60 min at room temperature in the presence of a horseradish peroxidase-conjugated goat anti-rabbit and anti-mouse secondary antibody (Pierce Biotechnology, Rockford, IL, USA) and visualized with a chemiluminescence kit (West Pico; Pierce Biotechnology, Rockford, IL, USA). Band intensities of the detected proteins were measured by densitometry.

### High performance liquid chromatography (HPLC) analysis of drug

We performed HPLC analysis to qualify the GEB extract used in this study. Samples of the GEB extract (1 mg) were dissolved in distilled water (1 mL). Samples were filtered and 10 μL of sample solution injected into the HPLC system (1260 infinity HPLC system, Agilent Technologies, Palo Alto, CA, USA) with a UV detector. Samples were analyzed on an Atlantis C18 analytical column (150 × 3.0 mm, 5 μm; Waters, Milford, MA, USA). The mobile phase consisted of distilled water (solvent A) and methanol (solvent B). The flow rate was 1 mL/min and gradient elution was used. The gradient elution program was as follows: initially 5% (v/v) solvent B, which was linearly increased to 70% (v/v) at 25 min and maintained up to 28 min. At 29 min, the composition of the mobile phase was returned to initial conditions, which were maintained for 11 min for column re-equilibration. The eluent was monitored at 221 nm.

### Statistical analysis

All procedures, assessments, and analyses were performed blindly to minimize observer bias. GraphPad Prism version 5 (GraphPad Software Inc., San Diego, CA, USA) was used for statistical analysis. All data are expressed as the mean ± SEM. Comparisons of total AIM scores and of biochemical or protein assays among groups were analyzed by a one-way ANOVA followed by a Bonferroni’s post-hoc test. In all of the analyses, differences were considered statistically significant at P < 0.05.

## Results

### Component analysis of the GEB extract

**Figure 2:**
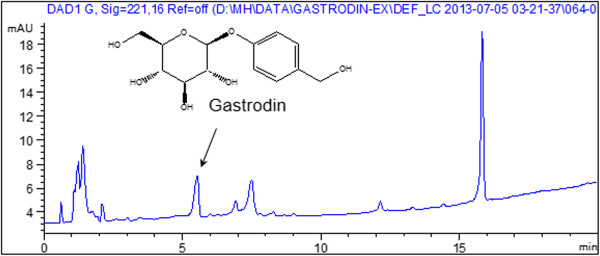
**A representative chromatogram for standardization of the Gastrodia elata Blume (GEB) extract sample.** The X-axis shows retention time; the Y-axis shows UV absorbance. Arrow indicates peak of gastrodin.

Gastrodin is known to be the one of main biologically active components of GEB. Thus, we sought to detect the main component of the GEB extract by HPLC analysis. Gastrodin was found at 5.6 min in the HPLC chromatogram. The content of gastrodin in the GEB extract was determined to be 0.237 ± 0.002%. A representative HPLC chromatogram of the Gastrodia elata Blume extract sample is shown in Figure 2.

### Effect of the GEB extract on L-DOPA-induced total AIMS score

**Figure 3:**
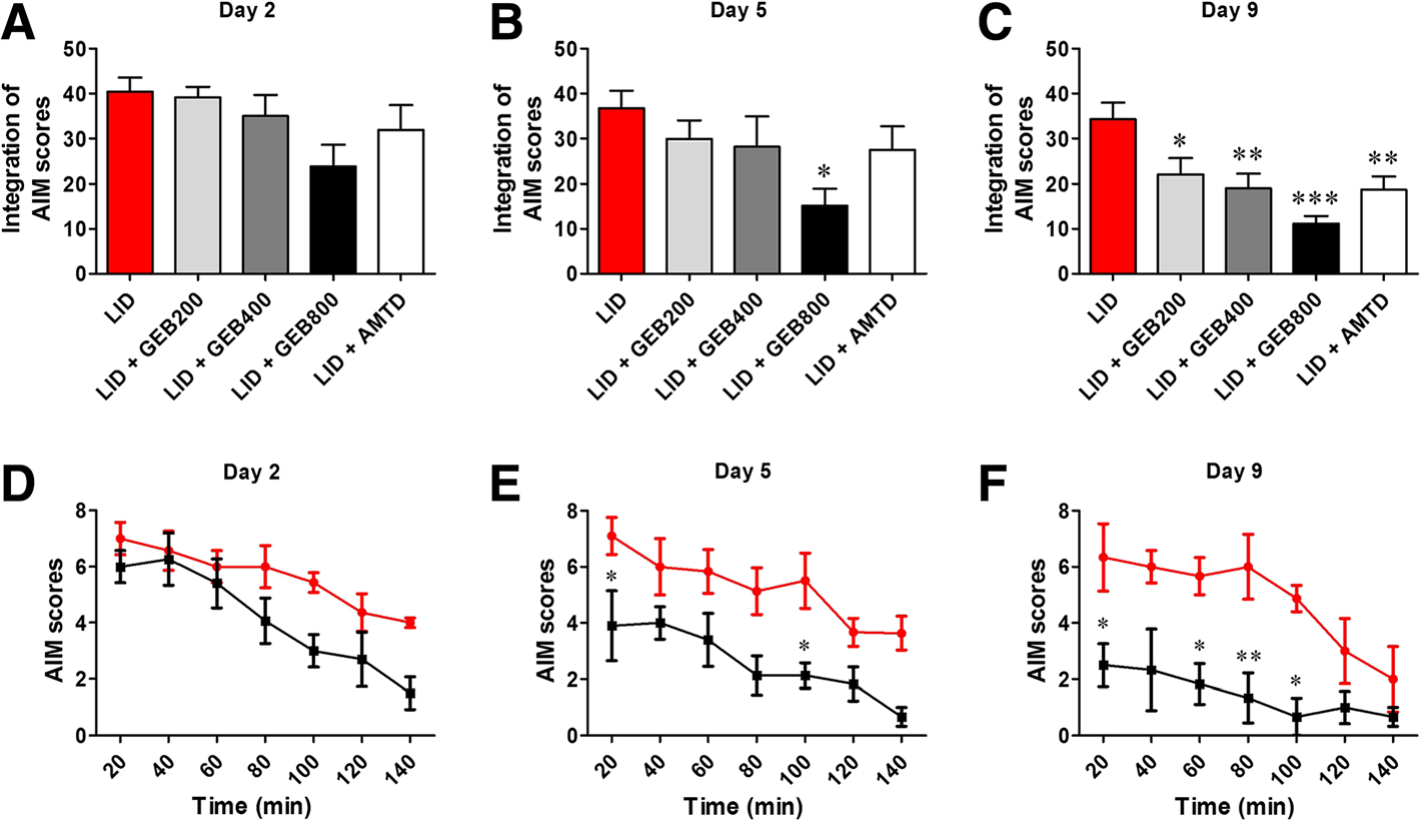
**Effect of the GEB extract on L-DOPA-induced abnormal involuntary movements (AIMs) in a PD mouse model bearing 6-OHDA lesions. (A-C)** Total AIMs were scored every 20 min over a 140 min period following L-DOPA injection. The integrated total AIMs scores on days 2 **(A)**, 5 **(B)**, and 9 **(C)** are shown. *P < 0.05, **P < 0.01, and ***P < 0.001 compared with the LID group. **(D-F)** The integrated temporal profiles of total AIMs scores on days 2 **(D)**, 5 **(E)**, and 9 **(F)** of the LID and LID + GEB800 treated group are shown. *P < 0.05 and **P < 0.01 compared with the LID group.

We found that various doses of the GEB extract and AMTD significantly lowered integrated total AIMs scores compared to the LID group on days 5 and 9 (Figure 3). On day 2, L-DOPA-induced AIMs scores were high and no significant differences were found between groups (40.4 ± 3.1, 39.1 ± 2.3, 35.0 ± 4.7, 23.9 ± 4.9, and 31.9 ± 5.6; the LID, GEB200, GEB400, GEB800, and AMTD groups, respectively; Figure 3A). On day 5, the L-DOPA group also exhibited high AIMs scores (36.7 ± 3.9), while all other treatment groups showed significantly lower AIMs scores compared to the LID group (29.9 ± 4.2, 28.3 ± 6.7, 15.1 ± 3.8, and 27.6 ± 5.1; the GEB200, GEB400, GEB800, and AMTD groups, respectively; F_6,41_ = 26.91, P < 0.0001; Figure 3B). The differences between AIMs scores became more pronounced on day 9 (34.3 ± 3.7; LID, 22.1 ± 3.6, 19.0 ± 3.7, 11.1 ± 1.7, and 18.7 ± 3.0; the GEB200, GEB400, GEB800, and AMTD groups, respectively; F_6,41_ = 31.54, P < 0.0001). A time point comparison between days 5 and 9 showed a significant decrease in total AIMs scores in the GEB800 group compared to the LID group (Figure 3D, E, and F).

### Effect of the GEB extract on four subtypes of AIM score induced by L-DOPA

**Figure 4:**
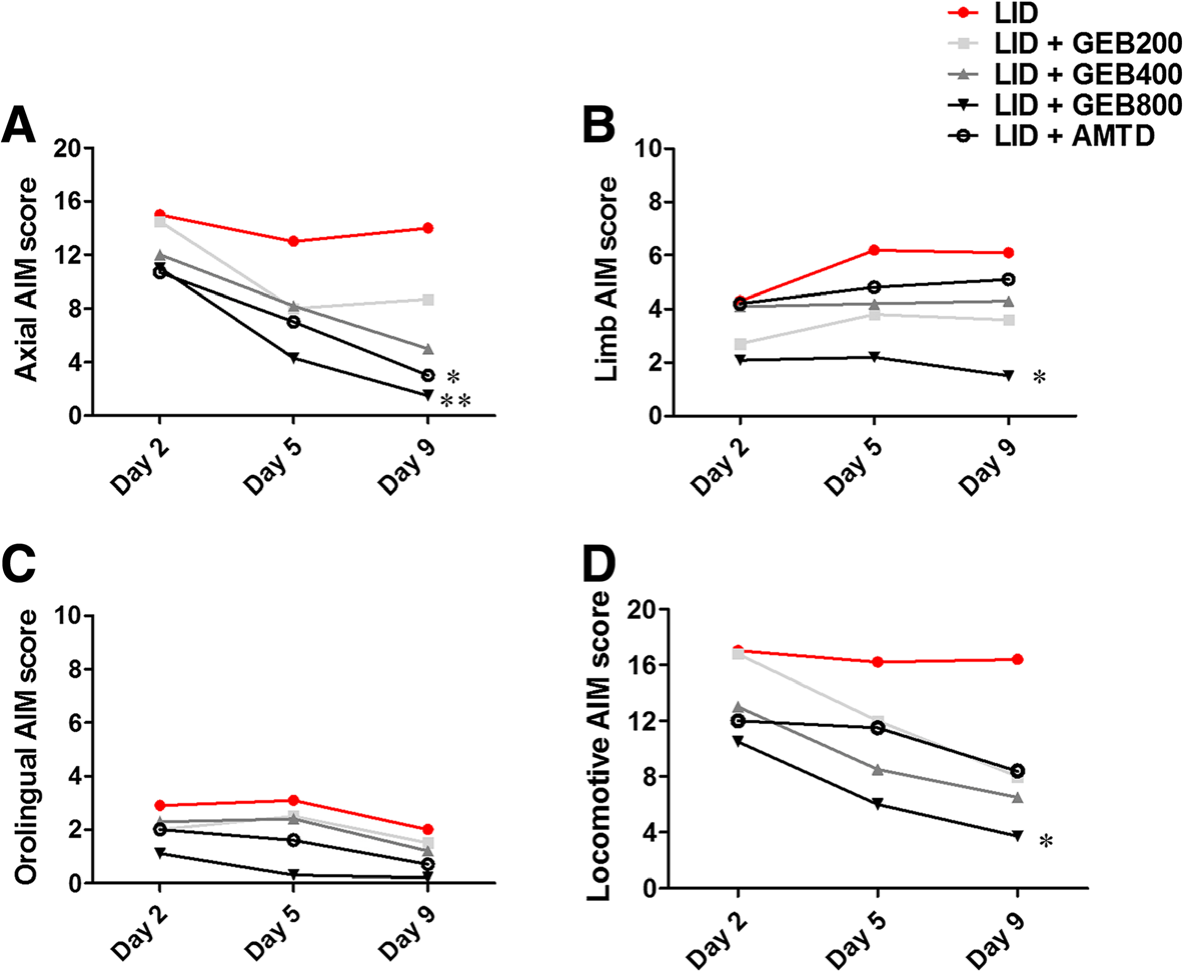
**The effect of GEB extract treatment on each subtype of axial, limb, and orolingual abnormal involuntary movement (AIM) induced by L-DOPA in a PD mouse model bearing 6-OHDA lesions. (A)** Axial AIM, **(B)** limb AIM, **(C)** orolingual AIM, and **(D)** locomotive AIM scores were measured on days 2, 5, and 9 after the initial L-DOPA injection. AIM scores were assessed every 20 min over 140 min following L-DOPA administration and were then integrated. *P < 0.05 and **P < 0.01 compared with the LID group.

In each integrative AIM subtype analysis, we found that the GEB400, GEB800, and AMTD groups showed decreased L-DOPA-induced axial, limb, orolingual, and locomotive AIMs compared to the LID group on day 9 (Figure 4).

### Effects of the GEB extract on LID-induced abnormal increase of phospho-ERK1/2 and FosB

**Figure 5:**
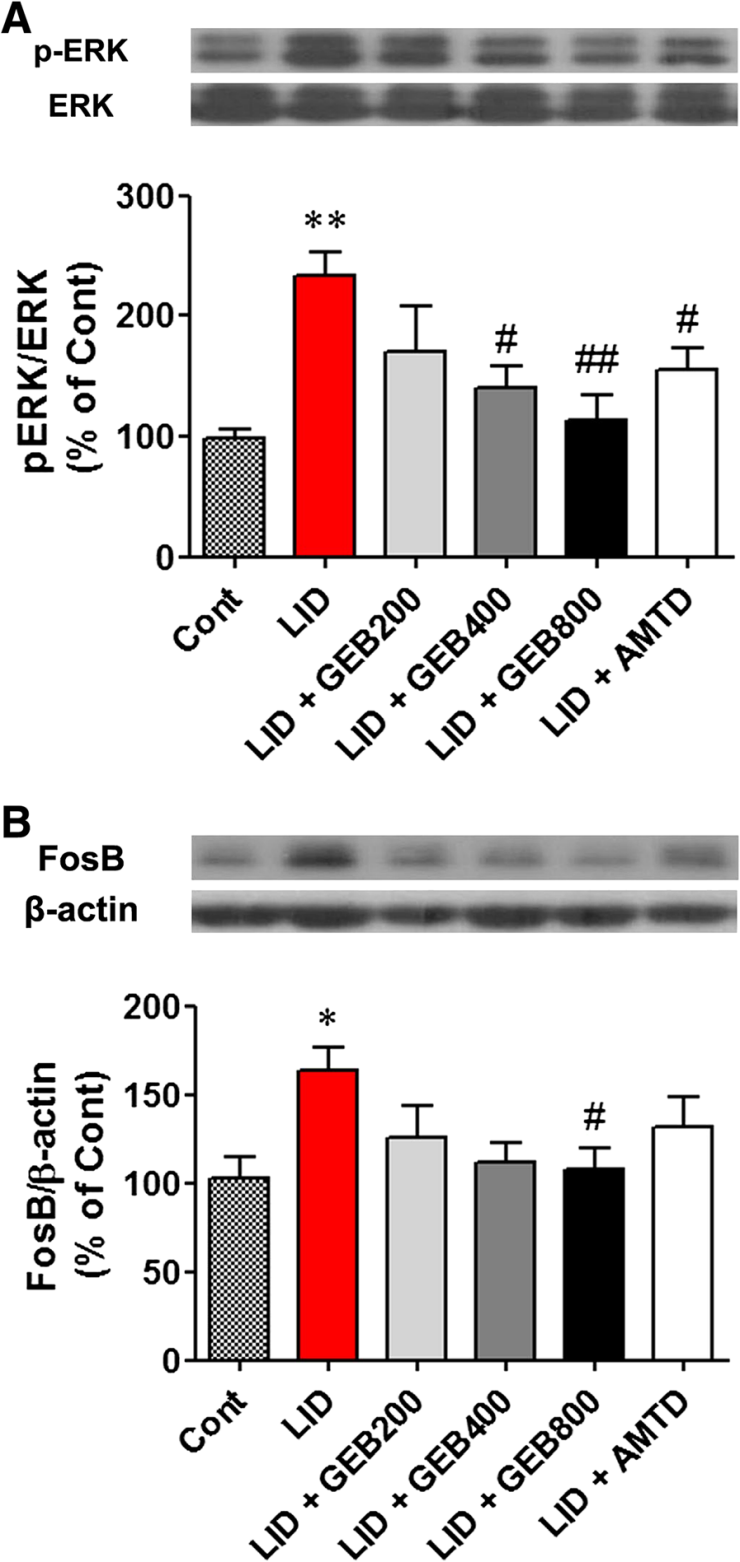
**Protein analysis of ERK1/2 phosphorylation and FosB expression in striatal tissue.** Protein levels were evaluated by western blotting the striatal extracts from each group of mice. **(A, B)** Phospho-ERK1/2 **(A)** and FosB **(B)** signals were observed in the ipsilateral sides of striata. Representative bar graph showing optical densities from the blotting experiments. *P < 0.05 and **P < 0.01 compared with the 6-OHDA control (Cont) group. #P < 0.05 and ##P < 0.01 compared with the LID group.

After the experiment, striatal tissues of the mice were prepared for protein analysis. Levels of phospho-ERK1/2 and FosB in the striatum were examined. The phosphorylation ratio of ERK1/2 was increased significantly in the striata of mice in L-DOPA group and was found to be 224% of the control (P < 0.01, compared to 6-OHDA control group). However, in the GEB200, GEB400, and GEB800 groups ERK1/2 phosphorylation was normalized to 171%, 142%, and 117% of the control, respectively (P > 0.05, P < 0.05, and P < 0.01). The AMTD group showed reduced ERK1/2 phosphorylation at approximately 162% of the control (Figure 5A).

FosB levels in the striata of mice in the L-DOPA group were significantly higher than in those of the 6-OHDA group (100% vs. 167% of control, P < 0.05). However, the GEB200, GEB400, and GEB800 groups showed decreased FosB levels at 128%, 115%, and 108% of the control, respectively (P > 0.05, P > 0.05, and P < 0.05). The AMTD group exhibited normalized FosB expression at 136% of control (Figure 5B).

## Discussion

Treatment with L-DOPA can lead to great enhancements in motor function for PD patients, but it also has serious adverse effects. Thus, researchers have sought to find novel therapies that mitigate the adverse effects of L-DOPA while enhancing its benefits. In the present study, we show that the GEB extract alleviates LID in a 6-OHDA-induced hemi-Parkinsonian mouse model. We have investigated its possible mechanism of action through the inhibition of ERK1/2 and FosB, which are abnormally activated due to chronic L-DOPA treatment. The GEB extract showed significant alleviation of dyskinesia in comparison with the anti-dyskinetic effect of AMTD, a drug used in the treatment of both PD and LID.

We found that high doses of GEB showed significant alleviation of LID in the PD mice model. GEB is used in a traditional Korean medicine for the treatment of abnormal activation in neuronal disorders, such as ischemia, dementia, and tremors. Previous *in vitro* and animal studies have described the effects of GEB in various neurological diseases. However, this is the first study to show that GEB exerts an anti-dyskinetic effect in an LID mouse model. Moreover, high doses of GEB (800 mg/kg) attenuated dyskinesia more effectively than did the marketed drug amantadine. Next, we analyzed the components of GEB to elucidate the mechanism behind this beneficial effect.

Previous researchers investigating the pathophysiology of LID have suggested that this condition arises from sensitization of the neuronal dopamine D1-like receptor and abnormal over-plasticity of the glutamate receptor, which induce over-excited signaling in the basal ganglia system. These authors reported that abnormal activation of proteins in the postsynaptic pathway induces abnormal dyskinesia in LID animal models. The proteins implicated in these studies include the dopamine- and cAMP-regulated neuronal phosphoprotein of 32 kDa (DARPP-32), ERK1/2, and transcription of the immediate early gene FosB [27,31,32]. Moreover, the pharmacological blockage of ERK1/2 signaling by a specific inhibitor or transgenic deletion of DARPP-32 showed greatly decreased LID behavior in the same mouse model [27].

The phenolic glucoside gastrodin has been used in traditional medicine for the treatment of various diseases and considered as a main bioactive component of GEB in the previous researches. Recent studies have shown gastrodin to have efficacy against oxidative stress [33,34], inflammation [35-37], obesity [38], memory deficiency [39,40], and Parkinson’s disease [23]. In these studies, the activity of gastrodin was linked to modulation of various cellular signaling pathways. Gastrodin was also effective in a mouse model of Parkinson’s disease. Gastrodin has also been found to inhibit abnormal NO synthase activity. It has also been observed that gastrodin inhibits cytokine and MAPK signaling expression induced by inflammation in BV-2 cells [41] and cardiomyocytes [42]. These studies showed that gastrodin inhibited abnormal increases in ERK1/2, JNK, and p38MAPK expression to reduce inflammation. Although these results give limited insight into the mechanistic link between gastrodin and the inhibition of the toxin-induced MAPK increase, they suggest the possibility that gastrodin may regulate the MAPK proteins to mitigate the effects of toxins or pathogens. In this study, abnormally increased ERK 1/2 phosphorylation in the LID mouse model was correlated with the increased expression of FosB. FosB is an immediate early gene that requires ERK activity for its transcription in the mouse striatum [43]. Based on these results, we speculate that GEB attenuates MAPK signaling and FosB expression. This leads, in turn, to normalized neuronal dopamine D1-like receptor overplasticity and attenuated LID. However, this possibility needs more evidence to draw a conclusion, and there is a limitation to figure out exact mechanism of effects of GEB from the results of this study. Further experiment would be needed to investigate what exact mechanism is underlying the effects of GEB.

## Conclusion

In conclusion, in this study, we found that the GEB extract alleviated LID, a set of adverse events associated with L-DOPA administration. Furthermore, we suggest that GEB induces the inhibition of MAPK signaling, including ERK 1/2 phosphorylation and FosB expression. This is the first study to show the beneficial effects of the GEB extract against adverse events arising from treatment with L-DOPA. These novel findings regarding the effects and mechanism of the GEB extract on LID represent an important step in the treatment of PD.

## Abbreviations

GEB: Gastrodia elata Blume; LID: L-dopa-induced dyskinesia; 6-OHDA: 6-hydroxydopamine; AIM: Abnormal involuntary movement.

## Competing interests

The authors declare that they have no competing interest.

## Authors’ contributions

DAR and KSN conceived this study, performed the experiments and wrote the manuscript. HDH and LHJ designed the experiments. YHH performed the component analysis of GEB. PJY performed the behavioral experiments. JSH and KJP designed the experiments and analyzed the data. PSU and PHJ conceived and supervised this project and wrote the manuscript. All authors read and approved the final manuscript.

## Pre-publication history

The pre-publication history for this paper can be accessed here:

http://www.biomedcentral.com/1472-6882/14/107/prepub
